# Supplementary material for: Shareable artificial intelligence to extract cancer outcomes from electronic health records for precision oncology research
Source: Nat Commun. 2024 Nov 12;15:9787. doi: 10.1038/s41467-024-54071-x (PMC11557593; doi:10.1038/s41467-024-54071-x)
Supplement: Supplementary file 1 — Supplementary Information [file 41467_2024_54071_MOESM1_ESM.pdf]

Supplementary Material

Supplementary Note 1:

Prompt to Llama-3-70B to label imaging reports:

```
def extract_note_data(note, llama_model, tokenizer):

    import re
    note = re.sub(r'\n+', '\n', note)
    messages = [
        {'role':'system', 'content': """You are a clinical oncology
data abstraction bot.
        Your job is to review clinical text and extract key data
elements."""
        },
        {'role':'user', 'content': "Here is an imaging report: \n" +
note + "\n" + """Now, generate a structured abstract of key data
elements from the report.
        These elements include whether the report describes active
current cancer; whether it describes worsening cancer; whether it
describes improving cancer; and whether it describes metastases to
various specific organs.
        Your output should be formatted as JSON like this:
        {
          "note_describes_current_cancer": "*yes/no*",
          "note_describes_worsening_cancer": "*yes/no*",
          "note_describes_improving_cancer": "*yes/no*",
          "note_describes_brain_metastasis": "*yes/no*",
          "note_describes_liver_metastasis": "*yes/no*",
          "note_describes_lung_metastasis": "*yes/no*",
          "note_describes_lymph_node_metastasis": "*yes/no*",
          "note_describes_bone_metastasis": "*yes/no*",
          "note_describes_adrenal_metastasis": "*yes/no*",
          "note_describes_peritoneal_metastasis": "*yes/no*"
        }

        ONLY output the JSON dictionary with the exact field names as
specified (all lower case).  Do not provide any introductory,
concluding, or explanatory text.

    """}
    ]


    trunc_messages = []
    for message in messages:
```

```
        message['content'] =
tokenizer.decode(tokenizer.encode(message['content'])[1:7100])
        trunc_messages.append(message)

    response =
llama_model.create_chat_completion(messages=trunc_messages,
max_tokens=750, temperature=0.05)

    return response, response['choices'][0]['message']['content']
```

Supplementary Note 2:
Prompt to Llama-3-70B to label discharge summaries:

```
def extract_note_data(note, llama_model, tokenizer):

    import re
    note = re.sub(r'\n+', '\n', note)
    messages = [
        {'role':'system', 'content': """You are a clinical oncology 
data abstraction bot.
        Your job is to review clinical and and extract key data 
elements."""
        },
        {'role':'user', 'content': "Here is a clinical document: \n" + 
note + "\n" + """Now, generate a structured abstract of key data 
elements from the report.
        These elements include whether the report describes active 
current cancer; whether it describes worsening cancer; and whether it 
describes improving cancer.
        Your output should be formatted as JSON like this:
        {
          "note_describes_current_cancer": "*yes/no*",
          "note_describes_worsening_cancer": "*yes/no*",
          "note_describes_improving_cancer": "*yes/no*"
        }

        ONLY output the JSON dictionary with the exact field names as 
specified (all lower case).  Do not provide any introductory, 
concluding, or explanatory text.

    """}
    ]



    trunc_messages = []
    for message in messages:
        message['content'] = 
tokenizer.decode(tokenizer.encode(message['content'])[1:7100])
        trunc_messages.append(message)

    response = 
llama_model.create_chat_completion(messages=trunc_messages, 
max_tokens=750, temperature=0.2)

    return response, response['choices'][0]['message']['content']
```

Supplemental Table 1: Non-small cell Lung cancer - Model performance per the AUROC

	Imaging reports			Oncologist noteas		
Model training center	DFCI			DFCI		
Model evaluation center	DFCI		MSK	DFCI		MSK
Model type	DFCI-imaging-teacher	DFCI-imaging-student	DFCI-imaging-student	DFCI-medonc-teacher	DFCI-medonc-student	DFCI-medonc-student
Outcome						
Any cancer	0.97	0.96	0.98	0.98	0.98	0.95
Progression	0.96	0.96	0.97	0.97	0.97	0.91
Response	0.98	0.97	0.97	0.98	0.97	0.94
Brain metastasis	0.99	0.99	0.99			
Bone metastasis	0.98	0.98	0.99			
Adrenal metastasis	0.99	0.99	1.0			
Liver metastasis	0.99	0.99	0.99			
Lung metastasis	0.96	0.96	0.95			
Nodal metastasis	0.98	0.97	0.97			
Peritoneal metastasis	1.00	1.00	0.96			

Supplemental Table 2: Non-small cell lung cancer - Model performance per the area under the precision recall curve (AUPRC)

	Imaging reports					Oncologist notes				
Model training center	DFCI					DFCI				
Model evaluation center	DFCI			MSK		DFCI			MSK	
	Outcome prevalence			Outcome prevalence		Outcome prevalence			Outcome prevalence	
AUPRC by model type		DFCi-imaging-teacher	DFCI-imaging-student		DFCI-imaging-student		DFCi-medonc-teacher	DFCI-medonc-student		DFCI-medonc-student
Outcome										
Any cancer	0.53	0.97	0.96	0.65	0.99	0.81	0.91	0.99	0.90	0.99
Progression	0.22	0.89	0.89	0.30	0.94	0.20	0.91	0.90	0.16	0.74
Response	0.08	0.80	0.80	0.09	0.84	0.15	0.91	0.88	0.19	0.81
Brain metastasis	0.09	0.92	0.93	0.03	0.80					
Bone metastasis	0.13	0.90	0.89	0.15	0.93					
Adrenal metastasis	0.04	0.88	0.85	0.05	0.92					
Liver metastasis	0.06	0.90	0.91	0.05	0.94					
Lung metastasis	0.31	0.92	0.91	0.43	0.94					
Nodal metastasis	0.12	0.88	0.86	0.19	0.87					
Peritoneal metastasis	0.01	0.78	0.75	0.02	0.79					

Supplemental Table 3: Non-small cell lung cancer - Model performance per the best F1 score

	Imaging reports					Oncologist notes				
Model training center	DFCI					DFCI				
Model evaluation center	DFCI			MSK		DFCI			MSK	
	Outcome prevalence			Outcome prevalence		Outcome prevalence			Outcome prevalence	
Best F1 by model type		DFCi-imaging-teacher	DFCI-imaging-student		DFCI-imaging-student		DFCi-medonc-teacher	DFCI-medonc-student		DFCI-medonc-student
Outcome										
Any cancer	0.53	0.91	0.90	0.65	0.95	0.81	0.97	0.97	0.90	0.97
Progression	0.22	0.80	0.82	0.30	0.88	0.20	0.85	0.85	0.16	0.68
Response	0.08	0.77	0.74	0.09	0.77	0.15	0.86	0.83	0.19	0.76
Brain metastasis	0.09	0.90	0.87	0.03	0.81					
Bone metastasis	0.13	0.86	0.87	0.15	0.87					
Adrenal metastasis	0.04	0.80	0.80	0.05	0.91					
Liver metastasis	0.06	0.84	0.84	0.05	0.90					
Lung metastasis	0.31	0.85	0.84	0.43	0.86					
Nodal metastasis	0.12	0.82	0.78	0.19	0.82					
Peritoneal metastasis	0.01	0.76	0.72	0.02	0.82					

Supplemental Table 4: Colorectal cancer - Model performance per the AUROC

	Imaging reports			Oncologist notes		
Model training center	DFCI			DFCI		
Model evaluation center	DFCI		MSK	DFCI		MSK
Model type	DFCI-imaging-teacher	DFCI-imaging-student	DFCI-imaging-student	DFCI-medonc-teacher	DFCI-medonc-student	DFCI-medonc-student
Outcome						
Any cancer	0.97	0.97	0.99	0.99	0.99	0.96
Progression	0.96	0.95	0.98	0.96	0.94	0.93
Response	0.98	0.98	0.98	0.98	0.98	0.95
Brain metastasis	1.00	1.00	0.99			
Bone metastasis	0.99	0.99	0.99			
Adrenal metastasis	0.99	0.99	1.00			
Liver metastasis	0.98	0.98	0.99			
Lung metastasis	0.98	0.97	0.99			
Nodal metastasis	0.96	0.95	0.96			
Peritoneal metastasis	0.98	0.98	0.97			

Supplemental Table 5: Colorectal cancer - Model performance per the area under the precision recall curve (AUPRC)

	Imaging reports					Oncologist notes				
Model training center	DFCI					DFCI				
Model evaluation center	DFCI			MSK		DFCI			MSK	
	Outcome prevalence			Outcome prevalence		Outcome prevalence			Outcome prevalence	
AUPRC by model type		DFCi-imaging-teacher	DFCI-imaging-student		DFCI-imaging-student		DFCi-medonc-teacher	DFCI-medonc-student		DFCI-medonc-student
Outcome										
Any cancer	0.52	0.97	0.97	0.76	1.00	0.69	1.00	0.99	0.74	0.98
Progression	0.23	0.88	0.88	0.40	0.97	0.18	0.86	0.82	0.16	0.70
Response	0.06	0.88	0.88	0.14	0.92	0.06	0.75	0.79	0.95	0.73
Brain metastasis	0.03	0.96	0.98	0.01	0.85					
Bone metastasis	0.04	0.85	0.84	0.07	0.94					
Adrenal metastasis	0.03	0.82	0.75	0.03	0.95					
Liver metastasis	0.21	0.93	0.95	0.43	0.98					
Lung metastasis	0.14	0.92	0.89	0.35	0.96					
Nodal metastasis	0.11	0.72	0.68	0.24	0.83					
Peritoneal metastasis	0.07	0.80	0.79	0.14	0.89					

Supplemental Table 6: Colorectal cancer - Model performance per the best F1 score

	Imaging reports					Oncologist notes				
Model training center	DFCI					DFCI				
Model evaluation center	DFCI			MSK		DFCI			MSK	
	Outcome prevalence			Outcome prevalence		Outcome prevalence			Outcome prevalence	
Best F1 by model type		DFCi-imaging-teacher	DFCI-imaging-student		DFCI-imaging-student		DFCi-medonc-teacher	DFCI-medonc-student		DFCI-medonc-student
Outcome										
Any cancer	0.52	0.93	0.93	0.76	0.97	0.69	0.98	0.97	0.74	0.95
Progression	0.23	0.83	0.81	0.40	0.91	0.18	0.80	0.77	0.16	0.70
Response	0.06	0.81	0.80	0.14	0.86	0.06	0.72	0.77	0.95	0.71
Brain metastasis	0.03	0.89	0.94	0.01	0.77					
Bone metastasis	0.04	0.75	0.79	0.07	0.89					
Adrenal metastasis	0.03	0.82	0.79	0.03	0.92					
Liver metastasis	0.21	0.92	0.92	0.43	0.94					
Lung metastasis	0.14	0.83	0.82	0.35	0.92					
Nodal metastasis	0.11	0.72	0.71	0.24	0.82					
Peritoneal metastasis	0.07	0.80	0.77	0.14	0.83					

Supplemental Table 7: Breast cancer - Model performance per the AUROC

	Imaging reports			Oncologist notes		
Model training center	DFCI			DFCI		
Model evaluation center	DFCI		MSK	DFCI		MSK
Model type	DFCI-imaging-teacher	DFCI-imaging-student	DFCI-imaging-student	DFCI-medonc-teacher	DFCI-medonc-student	DFCI-medonc-student
Outcome						
Any cancer	0.99	0.99	0.99	0.99	0.99	0.96
Progression	0.96	0.95	0.96	0.98	0.98	0.88
Response	0.98	0.98	0.96	0.97	0.94	0.91
Brain metastasis	0.99	1.00	0.98			
Bone metastasis	0.99	0.99	0.98			
Adrenal metastasis	0.99	0.99	1.00			
Liver metastasis	1.00	1.00	0.99			
Lung metastasis	0.99	0.99	0.97			
Nodal metastasis	0.99	0.99	0.98			
Peritoneal metastasis	0.99	0.99	0.97			

Supplemental Table 8: Breast cancer - Model performance per the area under the precision recall curve (AUPRC)

	Imaging reports					Oncologist notes				
Model training center	DFCI					DFCI				
Model evaluation center	DFCI			MSK		DFCI			MSK	
	Outcome prevalence			Outcome prevalence		Outcome prevalence			Outcome prevalence	
AUPRC by model type		DFCi-imaging-teacher	DFCI-imaging-student		DFCI-imaging-student		DFCi-medonc-teacher	DFCI-medonc-student		DFCI-medonc-student
Outcome										
Any cancer	0.70	1.00	0.99	0.86	1.00	0.89	1.00	1.00	0.77	0.98
Progression	0.27	0.90	0.90	0.42	0.95	0.14	0.93	0.90	0.15	0.64
Response	0.10	0.84	0.85	0.13	0.77	0.15	0.89	0.83	0.12	0.65
Brain metastasis	0.22	0.94	0.98	0.04	0.86					
Bone metastasis	0.34	0.95	0.97	0.56	0.98					
Adrenal metastasis	0.01	0.39	0.37	0.02	0.94					
Liver metastasis	0.13	0.94	0.97	0.35	0.98					
Lung metastasis	0.10	0.96	0.96	0.16	0.88					
Nodal metastasis	0.06	0.83	0.83	0.31	0.94					
Peritoneal metastasis	0.02	0.85	0.79	0.06	0.85					

Supplemental Table 9: Breast cancer - Model performance per the best F1 score

	Imaging reports					Oncologist notes				
Model training center	DFCI					DFCI				
Model evaluation center	DFCI			MSK		DFCI			MSK	
	Outcome prevalence			Outcome prevalence		Outcome prevalence			Outcome prevalence	
Best F1 by model type		DFCi-imaging-teacher	DFCI-imaging-student		DFCI-imaging-student		DFCi-medonc-teacher	DFCI-medonc-student		DFCI-medonc-student
Outcome										
Any cancer	0.70	0.97	0.96	0.86	0.98	0.89	0.99	0.99	0.77	0.95
Progression	0.27	0.84	0.83	0.42	0.88	0.14	0.86	0.85	0.15	0.60
Response	0.10	0.77	0.78	0.13	0.73	0.15	0.86	0.81	0.12	0.65
Brain metastasis	0.22	0.94	0.94	0.04	0.80					
Bone metastasis	0.34	0.95	0.93	0.56	0.94					
Adrenal metastasis	0.01	0.50	0.44	0.02	0.90					
Liver metastasis	0.13	0.91	0.93	0.35	0.95					
Lung metastasis	0.10	0.91	0.89	0.16	0.83					
Nodal metastasis	0.06	0.79	0.79	0.31	0.90					
Peritoneal metastasis	0.02	0.82	0.80	0.06	0.82					

Supplemental Table 10: Pancreatic cancer - Model performance per the AUROC

	Imaging reports			Oncologist notes		
Model training center	DFCI			DFCI		
Model evaluation center	DFCI		MSK	DFCI		MSK
Model type	DFCI-imaging-teacher	DFCI-imaging-student	DFCI-imaging-student	DFCI-medonc-teacher	DFCI-medonc-student	DFCI-medonc-student
Outcome						
Any cancer	0.96	0.96	0.99	0.83	0.81	0.98
Progression	0.92	0.93	0.98	0.97	0.96	0.94
Response	0.97	0.97	0.97	0.98	0.98	0.96
Brain metastasis	0.99	1.00	1.00			
Bone metastasis	0.99	0.99	1.00			
Adrenal metastasis	0.93	0.98	0.98			
Liver metastasis	0.95	0.95	0.99			
Lung metastasis	0.96	0.96	0.98			
Nodal metastasis	0.95	0.94	0.95			
Peritoneal metastasis	0.98	0.99	0.94			

Supplemental Table 11: Pancreatic cancer - Model performance per the area under the precision recall curve (AUPRC)

	Imaging reports					Oncologist notes				
Model training center	DFCI					DFCI				
Model evaluation center	DFCI			MSK		DFCI			MSK	
	Outcome prevalence			Outcome prevalence		Outcome prevalence			Outcome prevalence	
AUPRC by model type		DFCi-imaging-teacher	DFCI-imaging-student		DFCI-imaging-student		DFCi-medonc-teacher	DFCI-medonc-student		DFCI-medonc-student
Outcome										
Any cancer	0.58	0.97	0.97	0.75	1.00	0.82	0.94	0.93	0.81	1.00
Progression	0.24	0.82	0.83	0.37	0.96	0.13	0.79	0.77	0.16	0.79
Response	0.05	0.67	0.71	0.13	0.91	0.10	0.79	0.86	0.14	0.82
Brain metastasis	0.01	0.77	0.75	0.002	0.90					
Bone metastasis	0.07	0.81	0.81	0.05	0.96					
Adrenal metastasis	0.003	0.04	0.12	0.03	0.80					
Liver metastasis	0.25	0.82	0.83	0.33	0.99					
Lung metastasis	0.11	0.84	0.85	0.17	0.94					
Nodal metastasis	0.12	0.67	0.64	0.12	0.69					
Peritoneal metastasis	0.04	0.80	0.78	0.14	0.86					

Supplemental Table 12: Pancreatic cancer - Model performance per the best F1 score

	Imaging reports					Oncologist notes				
Model training center	DFCI					DFCI				
Model evaluation center	DFCI			MSK		DFCI			MSK	
	Outcome prevalence			Outcome prevalence		Outcome prevalence			Outcome prevalence	
Best F1 by model type		DFCi-imaging-teacher	DFCI-imaging-student		DFCI-imaging-student		DFCi-medonc-teacher	DFCI-medonc-student		DFCI-medonc-student
Outcome										
Any cancer	0.58	0.91	0.90	0.75	0.98	0.82	0.96	0.96	0.81	0.98
Progression	0.24	0.76	0.78	0.37	0.90	0.13	0.78	0.79	0.16	0.73
Response	0.05	0.58	0.63	0.13	0.86	0.10	0.80	0.84	0.14	0.76
Brain metastasis	0.01	0.80	0.75	0.002	0.88					
Bone metastasis	0.07	0.82	0.78	0.05	0.90					
Adrenal metastasis	0.003	0.08	0.22	0.03	0.78					
Liver metastasis	0.25	0.82	0.80	0.33	0.95					
Lung metastasis	0.11	0.76	0.78	0.17	0.89					
Nodal metastasis	0.12	0.71	0.67	0.12	0.70					
Peritoneal metastasis	0.04	0.81	0.79	0.14	0.81					

Supplemental Table 13: Prostate cancer - Model performance per the AUROC

	Imaging reports			Oncologist notes		
Model training center	DFCI			DFCI		
Model evaluation center	DFCI		MSK	DFCI		MSK
Model type	DFCI-imaging-teacher	DFCI-imaging-student	DFCI-imaging-student	DFCI-medonc-teacher	DFCI-medonc-student	DFCI-medonc-student
Outcome						
Any cancer	0.99	0.98	0.98	0.95	0.94	0.90
Progression	0.97	0.97	0.95	0.96	0.93	0.88
Response	0.96	0.97	0.96	0.95	0.94	0.89
Brain metastasis	0.93	0.99	0.99			
Bone metastasis	0.98	0.98	0.98			
Adrenal metastasis	NA*	NA*	1.00			
Liver metastasis	0.99	0.98	1.00			
Lung metastasis	0.99	0.99	0.98			
Nodal metastasis	0.99	0.99	0.96			
Peritoneal metastasis	1.00	1.00	0.96			

* NA, not applicable; metrics could not be calculated due to rarity of outcome in the cohort.

Supplemental Table 14: Prostate cancer - Model performance per the area under the precision recall curve (AUPRC)

	Imaging reports					Oncologist notes				
Model training center	DFCI					DFCI				
Model evaluation center	DFCI			MSK		DFCI			MSK	
	Outcome prevalence			Outcome prevalence		Outcome prevalence			Outcome prevalence	
AUPRC by model type		DFCi-imaging-teacher	DFCI-imaging-student		DFCI-imaging-student		DFCi-medonc-teacher	DFCI-medonc-student		DFCI-medonc-student
Outcome										
Any cancer	0.57	0.99	0.99	0.84	1.00	0.75	0.98	0.98	0.87	0.98
Progression	0.20	0.89	0.88	0.38	0.93	0.10	0.73	0.66	0.17	0.65
Response	0.03	0.56	0.57	0.11	0.79	0.07	0.59	0.45	0.11	0.60
Brain metastasis	0.007	0.23	0.39	0.02	0.77					
Bone metastasis	0.48	0.98	0.97	0.59	0.99					
Adrenal metastasis	NA*	NA*	NA*	0.02	0.90					
Liver metastasis	0.03	0.76	0.82	0.08	0.99					
Lung metastasis	0.02	0.84	0.77	0.10	0.89					
Nodal metastasis	0.09	0.92	0.91	0.37	0.90					
Peritoneal metastasis	0.002	1.0	1.00	0.02	0.74					

* NA, not applicable; metrics could not be calculated due to rarity of outcome in the cohort.

Supplemental Table 15: Prostate cancer - Model performance per the best F1 score

	Imaging reports					Oncologist notes				
Model training center	DFCI					DFCI				
Model evaluation center	DFCI			MSK		DFCI			MSK	
	Outcome prevalence			Outcome prevalence		Outcome prevalence			Outcome prevalence	
Best F1 by model type		DFCi-imaging-teacher	DFCI-imaging-student		DFCI-imaging-student		DFCi-medonc-teacher	DFCI-medonc-student		DFCI-medonc-student
Outcome										
Any cancer	0.57	0.96	0.95	0.84	0.97	0.75	0.93	0.93	0.87	0.95
Progression	0.20	0.83	0.84	0.38	0.85	0.10	0.71	0.70	0.17	0.63
Response	0.03	0.59	0.71	0.11	0.73	0.07	0.68	0.62	0.11	0.60
Brain metastasis	0.007	0.44	0.57	0.02	0.80					
Bone metastasis	0.48	0.96	0.95	0.59	0.96					
Adrenal metastasis	NA*	NA*	NA*	0.02	0.89					
Liver metastasis	0.03	0.79	0.86	0.08	0.96					
Lung metastasis	0.02	0.77	0.77	0.10	0.83					
Nodal metastasis	0.09	0.88	0.88	0.37	0.87					
Peritoneal metastasis	0.002	1.0	1.00	0.02	0.79					

* NA, not applicable; metrics could not be calculated due to rarity of outcome in the cohort.

Supplemental Table 16: Urothelial cancer - Model performance per the AUROC

	Imaging reports			Oncologist notes		
Model training center	DFCI			DFCI		
Model evaluation center	DFCI		MSK	DFCI		MSK
Model type	DFCI-imaging-teacher	DFCI-imaging-student	DFCI-imaging-student	DFCI-medonc-teacher	DFCI-medonc-student	DFCI-medonc-student
Outcome						
Any cancer	0.96	0.96	NA	0.97	0.97	NA
Progression	0.96	0.96	NA	0.92	0.90	NA
Response	0.97	1.00	NA	0.98	0.93	NA
Brain metastasis	1.00	1.00	NA			
Bone metastasis	0.98	0.98	NA			
Adrenal metastasis	0.99	0.99	NA			
Liver metastasis	0.99	0.99	NA			
Lung metastasis	0.96	0.95	NA			
Nodal metastasis	0.96	0.96	NA			
Peritoneal metastasis	0.98	0.98	NA			

NA, not applicable; no annotated data available for evaluation for this cohort

Supplemental Table 17: Urothelial cancer - Model performance per the area under the precision recall curve (AUPRC)

	Imaging reports					Oncologist notes				
Model training center	DFCI					DFCI				
Model evaluation center	DFCI			MSK		DFCI			MSK	
	Outcome prevalence			Outcome prevalence		Outcome prevalence			Outcome prevalence	
AUPRC by model type		DFCi-imaging-teacher	DFCI-imaging-student		DFCI-imaging-student		DFCi-medonc-teacher	DFCI-medonc-student		DFCI-medonc-student
Outcome										
Any cancer	0.44	0.96	0.95	NA	NA	0.82	0.99	0.99	NA	NA
Progression	0.28	0.89	0.88	NA	NA	0.26	0.82	0.76	NA	NA
Response	0.05	0.96	0.90	NA	NA	0.16	0.90	0.75	NA	NA
Brain metastasis	0.02	1.00	1.00	NA	NA					
Bone metastasis	0.16	0.92	0.91	NA	NA					
Adrenal metastasis	0.02	0.75	0.64	NA	NA					
Liver metastasis	0.04	0.85	0.83	NA	NA					
Lung metastasis	0.08	0.75	0.74	NA	NA					
Nodal metastasis	0.20	0.85	0.85	NA	NA					
Peritoneal metastasis	0.01	0.79	0.79	NA	NA					

NA, not applicable; no annotated data available for evaluation for this cohort

Supplemental Table 18: Urothelial cancer - Model performance per the best F1 score

	Imaging reports					Oncologist notes				
Model training center	DFCI					DFCI				
Model evaluation center	DFCI			MSK		DFCI			MSK	
	Outcome prevalence			Outcome prevalence		Outcome prevalence			Outcome prevalence	
Best F1 by model type		DFCi-imaging-teacher	DFCI-imaging-student		DFCI-imaging-student		DFCi-medonc-teacher	DFCI-medonc-student		DFCI-medonc-student
Outcome										
Any cancer	0.44	0.89	0.88	NA	NA	0.82	0.97	0.96	NA	NA
Progression	0.28	0.83	0.83	NA	NA	0.26	0.77	0.72	NA	NA
Response	0.05	0.93	0.90	NA	NA	0.16	0.86	0.78	NA	NA
Brain metastasis	0.02	1.00	1.00	NA	NA					
Bone metastasis	0.16	0.86	0.87	NA	NA					
Adrenal metastasis	0.02	0.75	0.63	NA	NA					
Liver metastasis	0.04	0.85	0.85	NA	NA					
Lung metastasis	0.08	0.75	0.79	NA	NA					
Nodal metastasis	0.20	0.82	0.83	NA	NA					
Peritoneal metastasis	0.01	0.86	0.86	NA	NA					

NA, Not applicable; no annotated data available for evaluation for this cohort

Supplemental Table 19: Renal cell cancer - Model performance per the AUROC

	Imaging reports			Oncologist notes		
Model training center	DFCI			DFCI		
Model evaluation center	DFCI		MSK	DFCI		MSK
Model type	DFCI-imaging-teacher	DFCI-imaging-student	DFCI-imaging-student	DFCI-medonc-teacher	DFCI-medonc-student	DFCI-medonc-student
Outcome						
Any cancer	0.99	0.99	NA	0.79	0.81	NA
Progression	0.96	0.95	NA	0.89	0.89	NA
Response	0.94	0.95	NA	0.95	0.85	NA
Brain metastasis	1.00	0.99	NA			
Bone metastasis	0.99	0.99	NA			
Adrenal metastasis	0.94	0.95	NA			
Liver metastasis	0.99	0.98	NA			
Lung metastasis	0.98	0.97	NA			
Nodal metastasis	0.99	0.99	NA			
Peritoneal metastasis	*	*	NA			

NA, not applicable; no annotated data available for evaluation for this cohort

* Outcome too rare to calculate metrics

Supplemental Table 20: Renal cell cancer - Model performance per the area under the precision recall curve (AUPRC)

	Imaging reports					Oncologist notes				
Model training center	DFCI					DFCI				
Model evaluation center	DFCI			MSK		DFCI			MSK	
	Outcome prevalence			Outcome prevalence		Outcome prevalence			Outcome prevalence	
AUPRC by model type		DFCi-imaging-teacher	DFCI-imaging-student		DFCI-imaging-student		DFCi-medonc-teacher	DFCI-medonc-student		DFCI-medonc-student
Outcome										
Any cancer	0.44	0.99	0.98	NA	NA	0.91	0.96	0.96	NA	NA
Progression	0.21	0.88	0.85	NA	NA	0.25	0.69	0.66	NA	NA
Response	0.04	0.38	0.54	NA	NA	0.15	0.82	0.58	NA	NA
Brain metastasis	0.06	0.98	0.97	NA	NA					
Bone metastasis	0.21	0.97	0.97	NA	NA					
Adrenal metastasis	0.05	0.59	0.60	NA	NA					
Liver metastasis	0.05	0.71	0.64	NA	NA					
Lung metastasis	0.11	0.82	0.78	NA	NA					
Nodal metastasis	0.16	0.92	0.96	NA	NA					
Peritoneal metastasis	*	*	*	NA	NA					

NA, not applicable; no annotated data available for evaluation for this cohort

* Outcome too rare to calculate metrics

Supplemental Table 21: Renal cell cancer - Model performance per the best F1 score

	Imaging reports					Oncologist notes				
Model training center	DFCI					DFCI				
Model evaluation center	DFCI			MSK		DFCI			MSK	
	Outcome prevalence			Outcome prevalence		Outcome prevalence			Outcome prevalence	
Best F1 by model type		DFCi-imaging-teacher	DFCI-imaging-student		DFCI-imaging-student		DFCi-medonc-teacher	DFCI-medonc-student		DFCI-medonc-student
Outcome										
Any cancer	0.44	0.93	0.94	NA	NA	0.91	0.99	0.98	NA	NA
Progression	0.21	0.78	0.78	NA	NA	0.25	0.73	0.71	NA	NA
Response	0.04	0.50	0.53	NA	NA	0.15	0.77	0.64	NA	NA
Brain metastasis	0.06	0.97	0.97	NA	NA					
Bone metastasis	0.21	0.95	0.94	NA	NA					
Adrenal metastasis	0.05	0.58	0.64	NA	NA					
Liver metastasis	0.05	0.76	0.72	NA	NA					
Lung metastasis	0.11	0.75	0.75	NA	NA					
Nodal metastasis	0.16	0.92	0.90	NA	NA					
Peritoneal metastasis	*	*	*	NA	NA					

NA, not applicable; no annotated data available for evaluation for this cohort

* Outcome too rare to calculate metrics
