# Supplementary material for: Shareable artificial intelligence to extract cancer outcomes from electronic health records for precision oncology research
Source: Nat Commun. 2024 Nov 12;15:9787. doi: 10.1038/s41467-024-54071-x (PMC11557593; doi:10.1038/s41467-024-54071-x)
Supplement: Supplementary file 3 — Reporting Summary [file 41467_2024_54071_MOESM3_ESM.pdf]

nature portfolio

Corresponding author(s): Kenneth L. Kehl

Last updated by author(s): October 29, 2024

Reporting Summary

Nature Portfolio wishes to improve the reproducibility of the work that we publish. This form provides structure for consistency and transparency in reporting. For further information on Nature Portfolio policies, see our Editorial Policies and the Editorial Policy Checklist.

Statistics

For all statistical analyses, confirm that the following items are present in the figure legend, table legend, main text, or Methods section.

n/a	Confirmed	
☐	☒	The exact sample size (*n*) for each experimental group/condition, given as a discrete number and unit of measurement
☒	☐	A statement on whether measurements were taken from distinct samples or whether the same sample was measured repeatedly
☒	☐	The statistical test(s) used AND whether they are one- or two-sided<br>*Only common tests should be described solely by name; describe more complex techniques in the Methods section.*
☒	☐	A description of all covariates tested
☒	☐	A description of any assumptions or corrections, such as tests of normality and adjustment for multiple comparisons
☐	☒	A full description of the statistical parameters including central tendency (e.g. means) or other basic estimates (e.g. regression coefficient) AND variation (e.g. standard deviation) or associated estimates of uncertainty (e.g. confidence intervals)
☒	☐	For null hypothesis testing, the test statistic (e.g. *F*, *t*, *r*) with confidence intervals, effect sizes, degrees of freedom and *P* value noted<br>*Give P values as exact values whenever suitable.*
☒	☐	For Bayesian analysis, information on the choice of priors and Markov chain Monte Carlo settings
☒	☐	For hierarchical and complex designs, identification of the appropriate level for tests and full reporting of outcomes
☒	☐	Estimates of effect sizes (e.g. Cohen's *d*, Pearson's *r*), indicating how they were calculated

Our web collection on statistics for biologists contains articles on many of the points above.

Software and code

Policy information about availability of computer code

Data collection	Data were collected in a Redcap database.
Data analysis	Model training and inference were performed using Pytorch, version 2.3.0. Code is available at github.com/prissmmnlp/dfci_msk_teacher_student_imaging and github.com/prissmmnlp/dfci_msk_teacher_student_medonc. Model weights are available at https://physionet.org/content/dfci-cancer-outcomes-ehr/1.0.0/ (authentication required).

For manuscripts utilizing custom algorithms or software that are central to the research but not yet described in published literature, software must be made available to editors and reviewers. We strongly encourage code deposition in a community repository (e.g. GitHub). See the Nature Portfolio guidelines for submitting code & software for further information.

Data

Policy information about availability of data

All manuscripts must include a data availability statement. This statement should provide the following information, where applicable:

- Accession codes, unique identifiers, or web links for publicly available datasets
- A description of any restrictions on data availability
- For clinical datasets or third party data, please ensure that the statement adheres to our policy

The DFCI medical records used to train teacher models, and to evaluate all models, contain protected health information and cannot be requested. Similarly, the MSK medical records used to evaluate student models contain private patient data and cannot be requested. The MIMIC data used to train student models are

available to credentialed researchers from Physionet at https://physionet.org/content/mimiciv/2.2/ . The numeric data used to generate the figures in this manuscript are provided as a supplemental file. Student model weights have been submitted to Physionet for publication to credentialed researchers; access will require the same process as access to the MIMIC data.

Research involving human participants, their data, or biological material

Policy information about studies with human participants or human data. See also policy information about sex, gender (identity/presentation), and sexual orientation and race, ethnicity and racism.

Reporting on sex and gender	Sex was defined based on institutional medical record systems.
Reporting on race, ethnicity, or other socially relevant groupings	Race and ethnicity were defined based on institutional medical record systems, as reported by patients when they registered for clinical care.
Population characteristics	Patient characteristics are described in Table 1 of the manuscript.
Recruitment	Patients were not specifically recruited for the current study. The cohort was defined based on patients at each institution whose tumors had already undergone tumor next-generation sequencing.
Ethics oversight	The Institutional Review Boards at DFCI and MSK approved collection of the data used in study.

Note that full information on the approval of the study protocol must also be provided in the manuscript.

Field-specific reporting

Please select the one below that is the best fit for your research. If you are not sure, read the appropriate sections before making your selection.

☒ Life sciences ☐ Behavioural & social sciences ☐ Ecological, evolutionary & environmental sciences

For a reference copy of the document with all sections, see nature.com/documents/nr-reporting-summary-flat.pdf

Life sciences study design

All studies must disclose on these points even when the disclosure is negative.

Sample size	Sample size was not prospectively defined specifically for this study; the analysis cohorts were derived from data collected through the AACR Project GENIE Biopharma Collaborative project.
Data exclusions	Analysis cohorts consisted of all patients with solid tumors that had undergone sequencing through our institutional precision medicine study, and who had imaging reports and/or oncologist notes for analysis. There were no additional exclusions.
Replication	The manuscript reports on external validation of the AI models developed at DFCI on data from MSK.
Randomization	N/A
Blinding	N/A

Reporting for specific materials, systems and methods

We require information from authors about some types of materials, experimental systems and methods used in many studies. Here, indicate whether each material, system or method listed is relevant to your study. If you are not sure if a list item applies to your research, read the appropriate section before selecting a response.

Materials & experimental systems

n/a	Involved in the study	
☒	☐	Antibodies
☒	☐	Eukaryotic cell lines
☒	☐	Palaeontology and archaeology
☒	☐	Animals and other organisms
☒	☐	Clinical data
☒	☐	Dual use research of concern
☒	☐	Plants

Methods

n/a	Involved in the study	
☒	☐	ChIP-seq
☒	☐	Flow cytometry
☒	☐	MRI-based neuroimaging

Plants

Seed stocks	N/A
Novel plant genotypes	N/A
Authentication	N/A
